# Antimicrobial resistance as a signature of soil restoration across a 143-year chronosequence

**DOI:** 10.1093/ismeco/ycag250

**Published:** 2026-09-02

**Authors:** Tim Goodall, Briony Jones, Amy C Thorpe, Robert I Griffiths, Hyun S Gweon, Daniel S Read, Richard Pywell, Susheel Bhanu Busi

**Affiliations:** UK Centre for Ecology & Hydrology, Benson Lane, Wallingford, OX10 8BB, United Kingdom; UK Centre for Ecology & Hydrology, Deiniol Road, Bangor, LL57 2UW, United Kingdom; UK Centre for Ecology & Hydrology, Benson Lane, Wallingford, OX10 8BB, United Kingdom; School of Environment, Bangor University, Bangor, LL57 2UR, United Kingdom; School of Biological Sciences, University of Reading, Reading, RG6 6UR, United Kingdom; UK Centre for Ecology & Hydrology, Benson Lane, Wallingford, OX10 8BB, United Kingdom; UK Centre for Ecology & Hydrology, Benson Lane, Wallingford, OX10 8BB, United Kingdom; UK Centre for Ecology & Hydrology, Benson Lane, Wallingford, OX10 8BB, United Kingdom

**Keywords:** antimicrobial resistance, soil health, chronosequence, soil restoration, metagenomics, biosynthetic gene clusters, fungal-bacterial ratio

## Abstract

Restoring agriculturally degraded habitats to species-rich grasslands is a vital conservation objective. During restoration, how the soil resistome matures alongside microbial community composition and function remains unclear. Here, we tested two competing hypotheses: whether the soil resistome co-occurs through a microbial structural maturation, in which soil restoration is associated with higher-order biotic interactions, or whether antimicrobial resistance (AMR) is instead associated with the competitive pressures and high bacterial taxonomic richness found in disturbed, eutrophic arable land. Using a unique land-use chronosequence on Salisbury Plain, UK, we investigated the trajectory of ecosystem reassembly following the cessation of agricultural activity. Our results demonstrate that AMR abundance increases significantly with restoration age, reaching a maximum in >143-year-old soils. The strongest predictor of this rise in AMR abundance was an increasing microbial eukaryotic signature rather than increasing microbial density, suggesting that resistome expansion is not associated with generalized spatial competition, but rather, co-occurs with structural maturation of the microbiome. We observed an order of magnitude increase in antibiotic biosynthetic potential, dominated by the emergence of streptomycin clusters. Microbial reorientation during soil maturation mirrors the expansion of a core resistome comprised of ancient, intrinsic mechanisms, such as Major Facilitator Superfamily (MFS) efflux pumps and RNA Polymerase-Binding Protein A (RbpA) target protection, in older soils. We demonstrate that endogenous AMR is a hallmark of healthy, restored soil ecosystems rather than a marker of anthropogenic soil degradation.

The recovery of plant communities during grassland restoration is comparatively well described [1–3], but the maturation of the below-ground microbiome and its resistome during soil recovery remains much less clear. In particular, there is a lack of certainty as to whether the soil resistome reflects disturbance [4], competition [5], and nutrient enrichment [6] in arable systems, or instead emerges as a feature of long-term ecological recovery [7]. This distinction matters because antimicrobial resistance (AMR) in soils is not solely a product of anthropogenic contamination [8] but is also rooted in ancient microbial interactions [9] and natural secondary metabolism [10]. Following our recent characterization of chronological soil functional maturation [11] using a multi-generational calcareous grassland restoration chronosequence on Salisbury Plain, UK, we here test how soil resistome composition and abundance change as soils mature from arable land, through restoration, to ancient grassland [12]. Grasslands were classified as arable (0 years; mean organic matter by Loss on Ignition (LOI) 10%), early regenerating (27 years; 16% LOI), regenerating (63 years; 20% LOI), and ancient (>143 years; 23% LOI) (Supplementary Table S1). By integrating metagenomic AMR profiles with microbial diversity, microbial biomass markers, eukaryote-to-prokaryote ratio, and biosynthetic gene cluster (BGC) potential (Supplementary Fig. S1, Supplementary  Methods  S1), we asked whether the soil resistome is associated with disturbed, species-rich arable soils, or with the functional maturation of restored ecosystems.

## Microbial reorientation, proliferation, and the resistome

**Figure 1:**
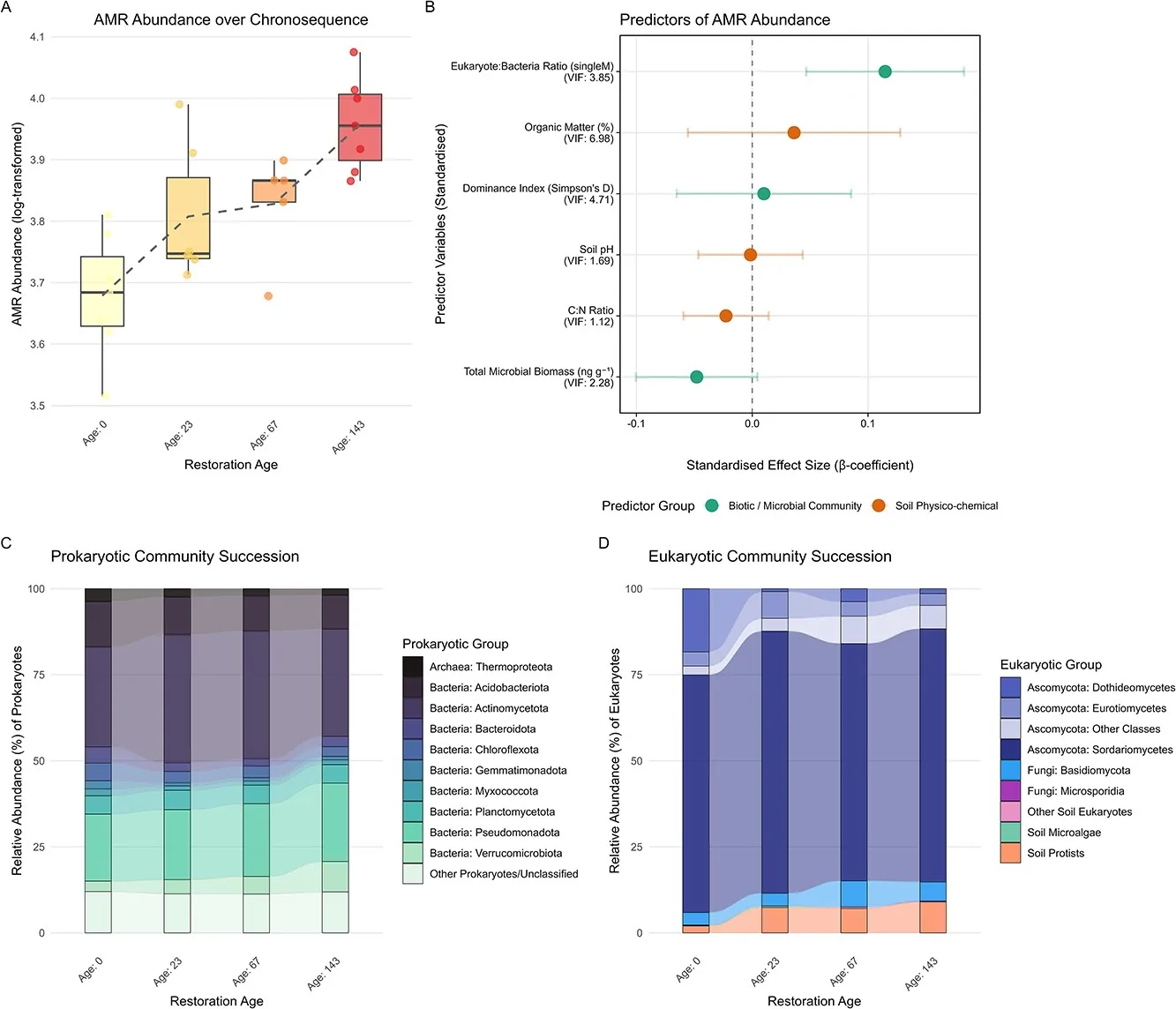
Soil resistome expansion, statistical predictors, and multi-kingdom taxonomic succession across the chronosequence; (A) AMR abundance over chronosequence: log-transformed total AMR gene abundance normalized to fragments per kilobase per million mapped reads on the chromosome (log_10_ FPKPMC +1) and mapped across the soil chronosequence; box plots indicate the median and interquartile range, individual points represent raw sample replicates (*n* = 24); the dashed trendline tracks the shift in group means over time; (B) drivers of AMR abundance: standardized effect size (*β*-coefficient) plot generated from a generalized least squares (GLS) multivariable model evaluating the direct predictors of soil AMR abundance; horizontal error bars signify 95% confidence intervals; biotic variables utilize low-variance inflation factor metrics, including total microbial biomass (ng g^−1^ dry soil) derived from microbial-specific phospholipid fatty acid extractions, and the Eukaryote:Bacteria ratio mapped via universal single-copy ribosomal marker genes (singleM); (C) prokaryotic community succession (SingleM): alluvial profile showing the relative abundance transitions (%) of dominant bacterial and archaeal phyla across the restoration timeline; (D) eukaryotic community succession (Kraken2): taxonomic succession of the soil eukaryotic community filtered to exclude macro-organism; fungal reads are partitioned into dominant classes of the *Ascomycota* phylum (*Sordariomycetes, Dothideomycetes, Eurotiomycetes*) alongside *Basidiomycota, Microsporidia*, and soil protists to visualize shifting trophic complexity.

Analysis of the metagenomic resistome across the soil chronosequence revealed a progressive accumulation of total AMR gene abundance over time (Fig. 1A, Supplementary Fig. S2). Rather than being associated with increasing microbial density, multivariable generalized least squares modelling demonstrated that resistome accumulation is strongly predicted by a shift in biotic equilibrium (Fig. 1B). When accounting for soil physico-chemical variations and community covariance, the most robust and significant positive predictor of total AMR gene abundance was the Eukaryote:Bacteria ratio (*β* = 0.115, *P* = .002), indicating that resistome expansion strongly coordinates with a shift in microbial balance. Other ecosystem features, including soil pH, organic matter, carbon-to-nitrogen ratio (C:N), microbial biomass, and prokaryotic Simpson’s dominance index, remained non-significant within the multivariable framework. The simultaneous increase of AMR and Eukaryote:Bacteria ratio is accompanied by successional transitions across major microbial lineages (Fig. 1C and D). Within the prokaryotic landscape, major phyla exhibit subtle relative shifts, including increases in Verrucomicrobiota alongside changes in key taxa such as Actinomycetota (Fig. 1C). Concurrently, the micro-eukaryotic community experiences a marked expansion in relative abundance of Soil Protists and *Basidiomycota* (which include key saprotrophic and mycorrhizal fungal guilds) at the expense of specific *Ascomycota* classes (Fig. 1D). Together, these taxonomic reorientations detail the multi-kingdom structural succession that co-occurs with resistome expansion across the chronosequence.

## Successional coupling of antibiotic biosynthetic potential and the resistome

**Figure 2:**
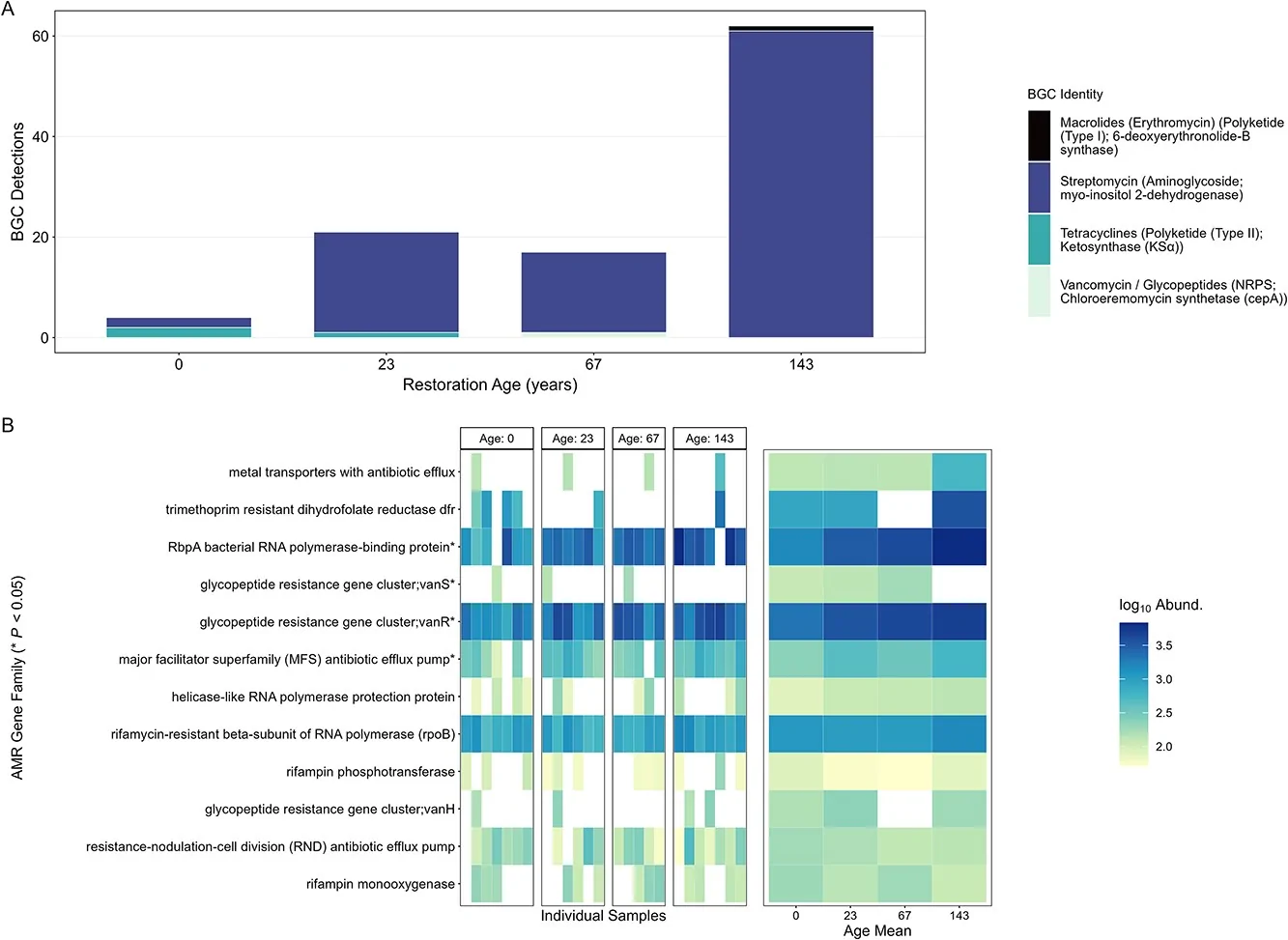
BGCs and resistome dynamics across the chronosequence; (A) stacked bar plot showing relative abundance and detection profiles of individual BGCs; (B) heatmaps tracking the dynamics of AMR gene families across individual samples (left panel) and their group-level mean log-transformed abundance per restoration age (right panel); asterisks (^*^) denote gene families exhibiting statistically significant linear abundance shifts across the chronosequence (*P* < .05), and only those with valid linear model slopes are displayed (see Supplementary Table S2 for full list of detected AMR gene families).

The successional, net increase in the resistome was matched by a 10-fold increase in genomic biosynthetic potential. To capture both canonical and novel secondary metabolic pathways, BGCs were predicted using an integrated pipeline combining antiSMASH v7.0 and GECCO. Predictions were consolidated into a non-redundant union based on overlapping genomic coordinates, ensuring each locus was quantified only once across tools (Supplementary Methods S1). Total BGC abundance increased by an order of magnitude from arable to ancient soils with unique gene clusters rising from a baseline of ~5 in arable soils to >50 in ancient grasslands (Fig. 2A). This substantial increase in biosynthetic potential is consistent with a plausible ecological basis for the parallel rise in endogenous AMR genes as BGCs and resistance genes are typically co-selected [13] (Fig. 2B; Supplementary Fig. S3). Resistome analysis across the chronosequence revealed successional increases in efflux systems (MFS, resistance-nodulation-division (RND), and ATP-binding cassette (ABC) transporters) and target protection mechanisms (*RbpA*) which safeguard essential cellular machinery from natural secondary metabolites [14], while anthropogenic marker genes, including the Class 1 integron integrase gene (*intI1*), sulfonamide resistance genes (*sul1, sul2*), disinfectant resistance genes (*qac*), and the human gut/sewage viral marker crAssphage remained below the limit of detection across all restoration sites (Supplementary Table S2). The enrichment of streptomycin-like BGCs in ancient soils suggests that mature systems harbour a more specialized secondary metabolic potential, a pattern hypothesized to reflect shifting spatial dynamics and ecological interactions between prokaryotic and eukaryotic communities [13].

## Conclusion

Our findings challenge the assumption that environmental AMR reflects anthropogenic degradation [8]. Across this 143-year chronosequence reflecting natural regeneration of calcareous grassland, resistome abundance increased alongside broader shifts in microbiome structure and biosynthetic potential, indicating that endogenous AMR can also be a feature of long-term ecological recovery. While primary succession in pristine glacier forelands exhibits early stabilization of resistance genes driven largely by mobile genetic elements (*intI1*) and horizontal gene transfer [7], our secondary succession model reveals a progressive, multi-century accumulation of intrinsic resistance mechanisms (e.g. MFS efflux, *RbpA*) that tracks with multi-kingdom structural maturation and BGC potential, with mobile element markers remaining below detection. These results highlight the importance of distinguishing natural, restoration-associated resistome signals from those linked to anthropogenic AMR pressure. We observed that clinical markers like rifampin monooxygenase remained static (Supplementary Fig. S3), while natural markers linked to soil BGCs were strongly enriched in mature sites, suggesting that AMR composition and abundance may provide a useful insight into microbiome reassembly and restoration status in calcareous grassland soils. To ensure that our findings, based upon measurements of genetic potential, are representative of active selections and interactions, metatranscriptomics and metabolomics will be required in future studies to definitively capture the phenotypic expression and metabolic kinetics associated with the observed relationships.

## Supplementary Material

Supplementary_material_ycag250

## Data Availability

Sequence data is available at NCBI-SRA under BioProject ID: PRJNA1424699. Scripts and data tables are available at doi:http://dx.doi.org/10.5281/zenodo.20396655.

## References

[1] Slodowicz D, Durbecq A, Ladouceur E, et al. The relative effectiveness of different grassland restoration methods: a systematic literature search and meta-analysis. *Ecol Solut Evid* 2023;4:e12221. 10.1002/2688-8319.12221

[2] Poniatowski D, Stuhldreher G, Helbing F, et al. Restoration of calcareous grasslands: the early successional stage promotes biodiversity. *Ecol Eng* 2020;151:105858. 10.1016/j.ecoleng.2020.105858

[3] Fagan KC, Pywell RF, Bullock JM, et al. Do restored calcareous grasslands on former arable fields resemble ancient targets? The effect of time, methods and environment on outcomes. *J Appl Ecol* 2008;45:1293–303. 10.1111/j.1365-2664.2008.01492.x

[4] Zeng Y, Feng R, Huang C, et al. Antibiotic resistance genes in agricultural soils: a comprehensive review of the hidden crisis and exploring control strategies. *Toxics* 2025;13:239. 10.3390/toxics13040239 PMC12031239 40278556

[5] Roy S, Dawson RA, Bradley JA, et al. Prevalence and dynamics of antimicrobial resistance in pioneer and developing Arctic soils. *BMC Microbiol* 2025;25:50. 10.1186/s12866-025-03745-7 PMC11771051 39871128

[6] Fu Y, Hu F, Wang F, et al. Field-based evidence for the prevalence of soil antibiotic resistomes under long-term antibiotic-free fertilization. *Environ Int* 2025;195:109202. 10.1016/j.envint.2024.109202 39681034

[7] Shen J-P, Li Z-M, Hu H-W, et al. Distribution and succession feature of antibiotic resistance genes along a soil development chronosequence in Urumqi No. 1 glacier of China. *Front Microbiol* 2019;10:1569. 10.3389/fmicb.2019.01569 PMC6629927 31354668

[8] Al-Khalaifah H, Rahman MH, Al-Surrayai T, et al. A one-health perspective of antimicrobial resistance (AMR): human animals and environmental health. *Life (Basel)* 2025;15:1598. 10.3390/life15101598 PMC12565658 41157271

[9] D’Costa VM, King CE, Kalan L, et al. Antibiotic resistance is ancient. *Nature* 2011;477:457–61. 10.1038/nature10388 21881561

[10] Perry EK, Meirelles LA, Newman DK. From the soil to the clinic: the impact of microbial secondary metabolites on antibiotic tolerance and resistance. *Nat Rev Microbiol* 2022;20:129–42. 10.1038/s41579-021-00620-w PMC8857043 34531577

[11] Goodall T, Busi SB, Jones B, et al. Taxonomic filtering accompanies functional expansion during long-term soil restoration. *ISME J* 2026;20. 10.1093/ismejo/wrag131 PMC13280953 42171661

[12] Redhead JW, Sheail J, Bullock JM, et al. The natural regeneration of calcareous grassland at a landscape scale: 150 years of plant community re-assembly on Salisbury Plain UK. *Appl Veg Sci* 2014;17:408–18. 10.1111/avsc.12076

[13] Surette MD, Wright GD. Lessons from the environmental antibiotic resistome. *Ann Rev Microbiol* 2017;71:309–29. 10.1146/annurev-micro-090816-093420 28657887

[14] Davies J, Davies D. Origins and evolution of antibiotic resistance. *Microbiol Mol Biol Rev* 2010;74:417–33. 10.1128/mmbr.00016-10 PMC2937522 20805405

