# Supplementary material for: Antimicrobial resistance as a signature of soil restoration across a 143-year chronosequence
Source: ISME Commun. 2026 Sep 2;6(1):ycag250. doi: 10.1093/ismeco/ycag250 (PMC13592167; doi:10.1093/ismeco/ycag250)
Supplement: Supplementary file 1 [file supplementary_material_ycag250.zip › amr_and_soil_age_260715_supp_meth_ycag250.docx]

***Supplementary Methods.***

*Study site, sampling and environmental characterisation*

Salisbury Plain, managed by the UK Ministry of Defence, covers approximately 38,000 ha and contains Western Europe’s largest remaining area of lowland calcareous grassland. Its long-term use as a military training area has limited agricultural intensification, creating a landscape suitable for examining grassland restoration chronosequences. Site selection followed the land-history classifications of Redhead^1^, derived from historic map series. In total, 25 sites were sampled: seven arable, six recent grassland, five middle-aged grassland and seven ancient grassland sites. These represented minimum unimproved grassland ages of 0 years for arable land, 23 years for recent grassland, 67 years for middle-aged grassland and 143 years for ancient grassland.

Soils were collected in January 2013. At each site, five subsamples were taken along a 100 m transect using 5 cm plastic corers to a depth of 15 cm, or until an impenetrable chalk horizon was reached. Soil below the organic horizon was homogenised, transported to the laboratory and stored at −20 °C.

Soil physicochemical analyses included pH, total and organic carbon, phosphorus, potassium, magnesium and texture, measured by NRM-Cawood Scientific. Soil moisture, organic matter by loss on ignition, total nitrogen and organic nitrogen were measured at the UKCEH Centralised Chemistry laboratories using established protocols. C:N ratios were calculated from organic carbon and organic nitrogen values.

*AMR profiling*

To profile the soil resistome, metagenomic reads were functionally annotated using a dual-pipeline approach. Specialised antimicrobial resistance genes (ARGs) were identified by querying non-redundant gene catalogues using ResScan (<https://github.com/hsgweon/resscan>), with the CARD database^2^. Simultaneously, broad functional categorisation was performed using eggNOG, mapping sequences to the eggNOG orthology database to capture intrinsic resistance determinants and associated metabolic pathways. To account for differences in sequencing depth and gene length, abundance was normalised to Fragments Per Kilobase per Million mapped reads onto the Chromosome (FPKPMC). This normalization provides a robust proxy for gene copy number per microbial cell by using the abundance of single-copy marker genes as a genomic denominator, allowing for direct quantitative comparisons of the resistome across the restoration chronosequence.

*Eukaryote-to-prokaryote ratio*

To quantify the relative abundance of eukaryotic and prokaryotic communities, we calculated the eukaryote-to-prokaryote ratio using domain-specific marker gene abundances from the SingleM pipeline^3^. SingleM utilizes a suite of 14 universal, single-copy ribosomal protein marker genes (e.g., rplB, rpsC) to estimate the relative cellular abundance of Bacteria, Archaea, and Eukaryotes. By using single-copy markers, this approach provides a robust proxy for the ratio of eukaryotic to prokaryotic cells, bypassing the biases typically associated with varying genome sizes and 16S/18S rRNA gene copy number variations across different microbial taxa.

*Microbial diversity*

SingleM marker-gene-based abundance estimates were used to produce prokaryotic abundance tables. For each sample alpha diversity values for Prokaryotic richness, Shannon diversity, and Simpson dominance were generated based on the counts obtained via SingleM.

Kraken2 read based assignments were used to examine micro-eukaryote community composition. Because environmental metagenomes frequently contain high proportions of genomic DNA from dominant macro-organisms (such as crop plants, transient macrofauna, and human handling contamination), a bioinformatic filtering pipeline was enforced to isolate the soil micro-eukaryotic signal through removal of all taxonomic assignments mapping to the Viridiplantae, Metazoa, and Hominidae.

*Microbial biomass*

To resolve absolute microbial community biomass, total lipid extracts obtained from fresh soil samples were structurally analysed using Gas Chromatography (GC)^4^. Raw peak profiles were filtered to isolate soil microbial signalling from confounding macro-organisms and plant inputs. High-order plant waxes and macrofauna structural lipids—specifically C20:0, C22:0, C24:0, C18:3ω6c, C18:3ω3c, C20:4ω6c, and C20:5ω3c—were excluded from baseline community metrics. The cumulative mass of all remaining active microbial phospholipid fatty acid (PLFA) fragments was summed to yield the total microbial lipid yield. Fungal markers - C18:1ω9c, C18:2ω6c, C18:2ω9t. Bacterial markers (Gram+ branched, Gram- monoenoics, cyclopropyl, actinomycetes) – “i-C15:0”, “a-C15:0”, “i-C:16:0”, “i-C17:0”, “a-C17:0”, “i-C18:0”, “a-C18:0”, “C16:1ω7c”, "C16:1ω9c", "C17:1ω10c", "C18:1ω11c", "C18:1ω11t", “cy-C17:0*”, “9,10-cy-C19:0”, “11,12-cy-C19:0”, "10Me-C16:0", "10Me-C17:0*", "10Me-C18:0*". Gravimentric measurements of soil moisture were used to correct the microbial yield to ng g^-1^ dry soil.

*Statistical Drivers of Resistome Accumulation (GLS Modeling)*

To evaluate the ecological and physico-chemical drivers of soil antibiotic resistome accumulation across the restoration chronosequence, we constructed a multivariable Generalized Least Squares (GLS) regression model using the nlme package in R. Total AMR abundance was log-transformed (log_10_ FPKPMC + 1) to satisfy the statistical assumptions of normality and homoscedasticity prior to model fitting.

*Predictor Variable Selection and Variance Inflation Adjustments*

The global model incorporated six environmental and biological predictors: soil pH, organic matter percentage (%), carbon-to-nitrogen ratio (C:N), Prokaryotic community dominance index (Simpson's D), absolute total microbial biomass (ng g^-1^ dry soil), derived from specific PLFA markers, and the Eukaryote-to-Bacteria ratio calculated via single-copy universal ribosomal marker genes (singleM).To prevent unstable parameter estimations driven by multicollinearity, a strict diagnostics pipeline was enforced using variance inflation factors (VIFs). Preliminary iterations containing highly collinear structural features (e.g., individual bacterial or fungal biomass yields) were iteratively refined. By substituting absolute fungal biomass with the relative Eukaryote:Bacteria ratio and absolute total microbial biomass, structural inflation was successfully mitigated. All final model predictors safely conformed to conservative threshold parameters: Soil pH (VIF = 1.69), C:N Ratio (VIF = 1.12), Total Microbial Biomass (VIF = 2.28), Eukaryote:Bacteria Ratio (VIF = 3.85), Dominance Index (VIF = 4.71), Organic Matter (VIF = 6.98). Standardised regression coefficients (*β*-coefficients) and associated 95% confidence intervals were extracted from the final stabilized GLS object to quantify the unique directional effect size of each predictor on resistome expansion while holding all other variables constant. Statistical significance was defined at alpha = 0.05.

*Biosynthetic gene clusters*

Biosynthetic Gene Clusters (BGCs) were identified and characterised using an integrated genomic mining pipeline. Metagenomic assemblies were processed through antiSMASH v7.0 (Antibiotic and Secondary Metabolite Analysis Shell)^5^ and GECCO (Gene Cluster Prediction with Conditional Random Fields)^6^ to ensure a comprehensive capture of both canonical and novel secondary metabolic pathways. To provide high-resolution functional annotation, predicted clusters were cross-referenced against the MIBiG (Manual of Minimum Information about a Biosynthetic Gene)^7^ database. A cluster was considered a 'known match' if it exhibited high homology to experimentally characterized BGCs (e.g., >40% gene similarity). Total BGC potential was quantified as the non-redundant sum of clusters identified across all platforms, normalised to the total number of contigs >5kb to account for assembly fragmentation. Redundant BGC predictions across antiSMASH and GECCO were consolidated based on genomic coordinates, ensuring each locus was counted only once in the final quantitative analysis.

*Statistical Filtering and AMR Family Inclusion Criteria*

The gene families displayed in Figure 2, Panel B were selected through an automated, data-driven filtering pipeline. To be eligible for rendering, an ARG family was required to pass a strict variance threshold allowing for the computation of an independent ordinary least squares linear regression model across the temporal gradient (lm(log_abund ~ age)). Families characterised by near-zero variance, extreme rarity (present in < 3 samples), or static saturation profiles across the chronosequence return non-computable slope parameters and were programmatically excluded from the visualisation grid to preserve structural clarity. For a complete, unfiltered overview of all detected environmental and clinical ARG classes—including ubiquitous or ultra-low abundance beta-lactamases, aminoglycosides, tetracyclines, and sulfonamides that did not meet the regression variance requirements—see Supplementary Table 2.

*Data and code availability*

Sequence data is available at NCBI-SRA under BioProject ID: PRJNA1424699. Scripts and data tables are available at https://doi.org/10.5281/zenodo.20396655

## References

1	Redhead, J. W. *et al.* The natural regeneration of calcareous grassland at a landscape scale: 150 years of plant community re-assembly on Salisbury Plain, UK. *Applied Vegetation Science* **17**, 408-418 (2014). <https://doi.org/https://doi.org/10.1111/avsc.12076>

2	Alcock, B. P. *et al.* CARD 2023: expanded curation, support for machine learning, and resistome prediction at the Comprehensive Antibiotic Resistance Database. *Nucleic Acids Research* **51**, D690-D699 (2023). <https://doi.org/10.1093/nar/gkac920>

3	Woodcroft, B. J. *et al.* Comprehensive taxonomic identification of microbial species in metagenomic data using SingleM and Sandpiper. *Nature Biotechnology* (2025). <https://doi.org/10.1038/s41587-025-02738-1>

4	Goodall, T. *et al.* Taxonomic filtering accompanies functional expansion during long-term soil restoration. *The ISME Journal* (2026). <https://doi.org/10.1093/ismejo/wrag131>

5	Blin, K. *et al.* antiSMASH 7.0: new and improved predictions for detection, regulation, chemical structures and visualisation. *Nucleic Acids Research* **51**, W46-W50 (2023). <https://doi.org/10.1093/nar/gkad344>

6	Carroll, L. M. *et al.* Accurate &lt;em&gt;de novo&lt;/em&gt; identification of biosynthetic gene clusters with GECCO. *bioRxiv*, 2021.2005.2003.442509 (2021). <https://doi.org/10.1101/2021.05.03.442509>

7	Zdouc, Mitja M. *et al.* MIBiG 4.0: advancing biosynthetic gene cluster curation through global collaboration. *Nucleic Acids Research* **53**, D678-D690 (2025). <https://doi.org/10.1093/nar/gkae1115>
