# Supplementary material for: Antimicrobial resistance as a signature of soil restoration across a 143-year chronosequence
Source: ISME Commun. 2026 Sep 2;6(1):ycag250. doi: 10.1093/ismeco/ycag250 (PMC13592167; doi:10.1093/ismeco/ycag250)
Supplement: Supplementary file 1 [file supplementary_material_ycag250.zip › figure_supp_1_amr_workflow_ycag250.pdf]

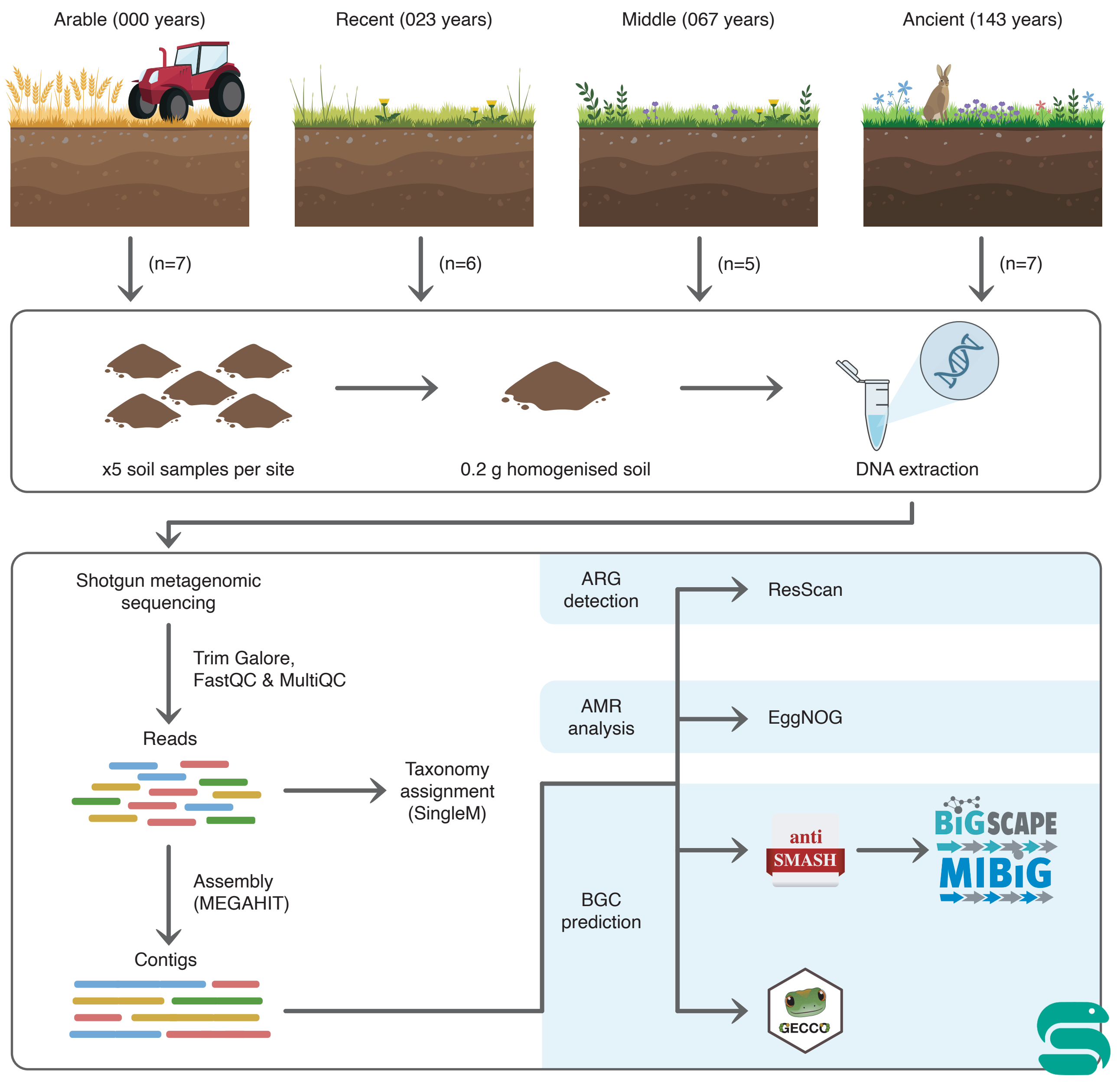
Arable (000 years)
Recent (023 years)
Middle (067 years)
Ancient (143 years)
(n=7)
(n=6)
(n=5)
(n=7)
x5 soil samples per site
0.2 g homogenised soil
DNA extraction
Shotgun metagenomic sequencing
Trim Galore, FastQC & MultiQC
Reads
Taxonomy assignment (SingleM)
Assembly (MEGAHIT)
Contigs
ARG detection
ResScan
AMR analysis
EggNOG
BGC prediction
anti SMASH
BiGSCAPE
MIBiG
GECCO
